# Towards Carbon Neutrality: The Impact of Renewable Energy Development on Carbon Emission Efficiency

**DOI:** 10.3390/ijerph182413284

**Published:** 2021-12-16

**Authors:** Feng Dong, Chang Qin, Xiaoyun Zhang, Xu Zhao, Yuling Pan, Yujin Gao, Jiao Zhu, Yangfan Li

**Affiliations:** School of Economics and Management, China University of Mining and Technology, Xuzhou 221116, China; chang.qin@cumt.edu.cn (C.Q.); ts18070012a31@cumt.edu.cn (X.Z.); TS21070029A31@cumt.edu.cn (X.Z.); ts19070137a31ld@cumt.edu.cn (Y.P.); TS20070030A31@cumt.edu.cn (Y.G.); ts20070019a31@cumt.edu.cn (J.Z.); yfl@cumt.edu.cn (Y.L.)

**Keywords:** renewable energy development, carbon emission efficiency, random forest regression, carbon neutrality

## Abstract

The energy transition and carbon emission efficiency are important thrust and target functions, respectively, for achieving carbon neutrality in the future. Using a sample of 30 Chinese provinces from 2006 to 2018, we measured their carbon efficiency using the game cross-efficiency data envelopment analysis (DEA). Then, a random forest regression model was used to explore the impact of renewable energy development on regional carbon emission efficiency. The results are as follows. First, China’s carbon emission efficiency in the southeast coastal area was better than that in the northwest area. Second, renewable energy development first inhibited and then promoted carbon emission efficiency, and there existed a reasonable range. Third, through a regional heterogeneity analysis, the trend of the influence of renewable energy development on carbon emission efficiency was found to not be significantly different in eastern, central, and western China, but there was a certain gap in the reasonable range. Our study not only helps to promote the study of renewable energy development and the carbon neutral target, but also provides an important reference for Chinese policy-makers to design a reasonable carbon emissions reduction path.

## 1. Introduction

Since the industrial revolution, the economy has grown rapidly, and people’s living standards have improved. However, this has been accompanied by a surge in energy consumption, which has led to a series of ecological and environmental problems [1,2]. In particular, carbon dioxide emissions have increased dramatically. This has caused the greenhouse effect, which has resulted in global warming, glacial melting, rising sea levels, and other climate issues that pose challenges to global sustainable development [3]. In response to the climate challenge, countries have joined the zero-carbon race. At present, 130 countries have set carbon neutrality targets to achieve net-zero carbon emissions by mid-century [4]. According to the Climate Change report [5] by the Intergovernmental Panel on Climate Change, the massive burning of fossil fuels is the main reason for the surge in carbon emissions. As highlighted by the International Energy Agency, the transition to renewable energy is the key to reaching the Paris Agreement’s global warming target of 2.0 °C. Various countries have also introduced policies to support the energy transition, such as the United States Department of Energy Research and Innovation Act, the European Union’s Strategic Energy Technology Plan, and Japan’s Fifth Energy Resources Basic Plan. Therefore, exploring the impact of renewable energy development on the economy and society has become a hot issue of academic and policy concern.

**Figure 1:**
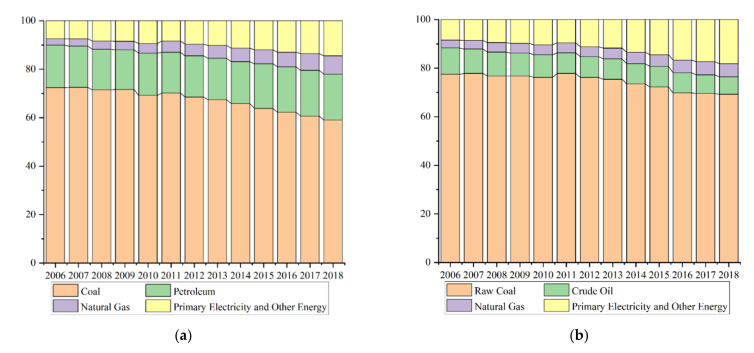
The energy structure in China. Note: the figure shows that China’s energy consumption structure and energy production structure were gradually optimized from 2006 to 2018. The share of fossil energy in China is over 80%, both on the production side and the consumption side. (**a**) Energy Consumption Structure. (**b**) Energy Production Structure.

China, the largest developing country, has experienced rapid growth since its reform and opening-up [6]. However, since 2007, China has become the world’s largest carbon emitter [7]. To achieve the voluntary reduction target and to transition to a low-carbon economy, China proposed to achieve carbon neutrality by 2060 [8]. With years of energy transition, China’s energy structure is gradually being optimized (as shown in Figure 1). However, China’s fossil energy consumption still accounts for more than 80% of the total energy consumption. It is generally recognized that reducing CO_2_ emissions is crucial to the achievement of sustainable development goals [9]. For developing countries such as China, the government must continue to improve the living standards of people. So, ensuring economic growth while achieving the carbon neutrality target has become an urgent issue in China’s ecological management. As an important evaluation criterion of a low-carbon economy, the improvement of the carbon emission efficiency (i.e., ecologically sensitive productivity with carbon emissions as an undesirable output) implies better coordination between economic growth and ecological development. Therefore, exploring the impact of renewable energy development on carbon emission efficiency can provide a basis for the decision making that is necessary to balance economic growth and carbon neutrality goals.

The energy transition remains urgent in China [10]. In recent years, China has witnessed the unprecedented development of the renewable energy industry [11]. It has been demonstrated that China’s renewable energy development has a significant positive relationship with carbon emissions in the long term, but not in the short term [12]. The impact of renewable energy development on carbon emissions is complex, with an inverted “U”-shaped trend overall [13]. Improving the carbon emission efficiency, which is one of the main ways to reduce carbon emissions, is influenced by many factors such as economic development [14] and environmental policies [15]. Therefore, to enhance the carbon emission efficiency, it is necessary to optimize the allocation of economic, environmental, and natural resources [16] and adjust the energy structure. As a clean and sustainable energy source, the development of renewable energy affects the traditional energy consumption structure and thus the carbon emission efficiency.

Developing renewable energy and promoting the energy transition have become ways to achieve the goal of carbon neutrality [17]. The socio-economic indicator for the bioeconomy (SEIB) is a new indicator by which to evaluate the performance of an energy transition, incorporating the bioeconomy sector into the measurement system, which is essential for achieving the energy transition [18]. Dialga [19] established a sustainable development evaluation system that provides policy-makers with different policy and strategy references. Stakeholders become complicated with the involvement of government, business, and the public. This makes the energy transition a complex and challenging ongoing process with an uncertain outcome [20]. In this context, Höfer and Madlener [21] derived detailed policy recommendations for a sustainable energy transition by considering multiple stakeholders.

All of the above illustrate the importance and challenge of the energy transition. Therefore, the development of renewable energy is particularly important. On the one hand, the development of renewable energy has many advantages. Increasing the amount of renewable energy can improve the level of sustainable development [22]. Moreover, a renewable energy policy can effectively reduce carbon emissions and optimize the energy structure [23]. On the other hand, there are disadvantages to developing renewable energy sources. Integrating renewable energy resources into the grid infrastructure is a challenging task [24] that has certain requirements for infrastructure development. In addition, the development of renewable energy needs economic support [25], that is, there is a threshold for the development of renewable energy. These produce resistance to the development of renewable energy sources.

In short, the development of renewable energy is full of opportunities and challenges. However, with the deterioration of the climate and the goal of carbon neutrality, energy transition has become a general trend. Exploring the relationship between renewable energy and carbon emission efficiency has become a research hotspot.

How does renewable energy affect carbon efficiency? To what extent does renewable energy affect carbon efficiency? What have been the results? To answer these questions, we carried out the following research work. First, a game cross-efficiency data envelopment analysis (DEA) that considers non-desirable outputs was used to measure the Chinese provincial carbon emission efficiency, which solves the alternate optimality problem. Second, through random forest regression, the importance and significance of renewable energy development to carbon emission efficiency were identified, and the marginal impact of renewable energy development on carbon emission efficiency was simulated. Third, 30 provinces were divided into three regions (eastern China, central China, and western China), and a regional heterogeneity analysis was conducted to obtain the results of the sub-regional analysis.

## 2. Literature Review and Research Gap

### 2.1. Carbon Emission Efficiency

The measurement of carbon efficiency has been extensively studied, and the mainstream methods include the carbon productivity measure, the carbon index method, stochastic frontier analysis (SFA), and data envelopment analysis. Among them, the carbon productivity measure (i.e., CO_2_ emissions divided by nominal GDP) is commonly used to evaluate whether a country meets the criteria for energy efficiency and emissions reductions [26]. The carbon index method (i.e., carbon emissions per unit of energy consumed) is commonly used to measure the extent to which developing countries contribute to energy conservation and emissions reductions [27]. SFA, which was first proposed by Meusen and Broeck [28], is a model whose most important feature is that the parameters of the frontier function and the technology inefficiency function can be estimated simultaneously. DEA, which was first proposed by Charnes and Cooper [29], measures effectiveness based on multiple input indicators and multiple output indicators using a linear programming approach [30]. Due to the non-efficiency, the term and the form of the function should be set in advance in SFA, which can easily cause bias in the results. Therefore, an increasing number of studies use DEA to evaluate carbon emission efficiency [31]. Numerous models have been derived based on DEA models, such as SBM-DEA [32], SE-SBM [33], the three-stage DEA [34], EBM-DEA [35], the global Malmquist–Luenberger index [36], the intermediate DEA [37], and the meta-frontier DEA [38]. With these models, studies have calculated the carbon emission efficiency as shown in Table 1.

In addition, the carbon emission efficiency among regions is also one of the hot spots of research. The research results show that the eastern region of China has the highest carbon emission efficiency; it is followed by the central region and, finally, the western region [38,43,44].

Research on the factors influencing carbon emission efficiency has received increasing attention. In terms of economic development, there are coupling effects between economic growth and carbon emission reduction efficiency in the long run and, in the short term, regions at different stages of economic development have different coupling effects [45]. Furthermore, the impact of the urbanization level on carbon efficiency shows an inverted “U” shape. The urbanization level has a positive impact at the beginning, but, as the urbanization level increases, it is detrimental to the development of carbon efficiency after it reaches a critical point [46]. In terms of energy, Ning et al. [47] demonstrated that the energy intensity has a negative impact on the carbon emission efficiency, which means that the more abundant the resource the lower the carbon emission efficiency [48], and when the energy efficiency is increased to a certain level, it can promote an increase in the carbon emission efficiency [49]. He et al. [50] showed that renewable energy technology innovation can have a positive impact on carbon efficiency, and its significance depends largely on the degree of local market segmentation and the market potential.

### 2.2. Relevant Studies on Renewable Energy Development

Over the past decade, international sources of renewable energy have continued to grow. Renewable energy sources, such as wind, solar, hydro, biomass, and geothermal energy, have taken their place in the global energy supply [51]. Renewable energy plays an important role in sustainable development in the context of a deteriorating environment [52].

Currently, research on renewable energy is focused on both the economic and environmental aspects [53]. First, on the relationship between renewable energy development and economic growth, Shahbaz et al. [54] found that renewable energy consumption has a positive impact on economic development in most countries, but it has a significant negative impact in countries where renewable energy consumption is in its infancy. Further studies have shown that there is a threshold effect on the impact of renewable energy on economic development. In countries with low levels of renewable energy utilization, renewable energy consumption can have a negative impact on economic growth, but when renewable energy development exceeds a certain threshold, it can bring about positive economic benefits [55]. Second, regarding the relationship between renewable energy development and environmental pollution, Zhao et al. [56] showed that the development of renewable energy can effectively mitigate continuous environmental degradation. Moreover, renewable energy consumption can curb carbon emissions and improve the environment’s quality significantly [57]. The increased level of renewable energy development in China is conducive to the reduction of carbon emissions, and the emissions reduction effect of renewable energy is becoming more obvious each year [58]. Overall, the use and expansion of renewable energy sources are both economically and ecologically beneficial [59].

In summary, there are sufficient studies on carbon emission efficiency and renewable energy development to provide a theoretical basis for studying the impact of renewable energy on carbon emission efficiency. However, there are still some shortcomings. First, in terms of the research object, the existing literature mainly focuses on the impact of renewable energy development on economic growth, carbon emissions, and environmental pollution, whereas few studies have focused on the renewable energy–carbon emission efficiency relationship. Second, the existing literature on carbon emission efficiency measurement is mostly based on the self-assessment of decision units, but it ignores the non-cooperative game among the decision units, which leads to over-estimated measurement results and cannot compare effective decision units (with an efficiency value of 1) in a hierarchical manner. Third, in the empirical strategy, traditional measures based on mean regression are mostly used, while the heterogeneous marginal effects with variations in variables are not available.

To fill these gaps, this paper contributes to the literature in the following three respects. First, the game cross-efficiency DEA that combines self-assessment and other assessment methods was used to measure the carbon emission efficiency of 30 Chinese provinces, which considers the game and competition relationship among the 30 provinces. Second, random forest regression was used to depict the curve of the marginal effect of renewable energy development on the carbon emission efficiency while maintaining the original characteristics of the data, which expands the research perspective. Third, a heterogeneity analysis was carried out according to the geographical location, and the optimal interval of each region for renewable energy development was simulated to provide a scientific reference for the development of renewable energy according to local conditions. This study provides empirical evidence and policy implications for China and other late-developing countries to balance their economic growth and carbon neutrality goals in the energy transition.

## 3. Methodology and Data

### 3.1. Methodology

#### 3.1.1. DEA Game Cross-Efficiency Model Considering Undesired Outputs

With the increasing research on energy and environmental issues, scholars have developed many models to measure the efficiency of carbon emissions. In this study, a DEA game cross-efficiency model considering undesired outputs was used as a method to solve for the carbon emission efficiency. This model, as an extension of the DEA cross-efficiency model, solves the alternate optima issue. This enables each DMU to pursue the efficiency maximization objective without worsening the efficiency of other DMUs [60].

#### 3.1.2. Random Forest Model

As one of the statistical learning methods, random forest is widely used in various fields. It has excellent prediction performance and few restrictions on the nature of the data. In addition, random forest methods can deal with not only classification problems but also regression problems. Combining its advantages, we used the random forest model to explore the impact of renewable energy development on carbon emission efficiency.

(1) Decision tree

The part that makes up the random forest is the decision tree, which is a tree model describing the classification or regression and consists of branches and nodes, with the leaf nodes representing the final classification results. When the output variable (the explained variable) of a decision tree is a continuous variable, it is called a regression tree. Regression trees can be built without satisfying many assumptions of classical regression, and the fit of regression tree models is generally better than that of linear regression models.

A decision tree is of the form h(x,Θ), where x is the input vector and Θ is the random vector whose character and dimensionality depend on the use of the decision tree.

(2) Bagging

Bagging refers to random repetitive sampling by bootstraps to generate a training set with variables and samples that may or may not be duplicated. The classifier is repeatedly trained by these training sets that do not use all the data, and, eventually, multiple classifiers are obtained. Considering that the accuracy of individual classifiers is not significant, a voting method is used, and the one with the most votes is used as the result of the final classification. When used for regression, the results of the classifiers are averaged to obtain the regression outcomes.

(3) Random forest

Random forest can be seen as a combination of decision trees and bagging. First, a part of the original data set is randomly selected as the training set, then a new subset is randomly selected from the training set to build multiple decision trees with the same distribution. The final result is obtained by voting or averaging. It can improve the accuracy of the model by generating multiple decision models and aggregating the decision results. It can efficiently handle a large number of input variables in the process of making decisions. Random forest is also able to assess the importance of variables, which allows the selection of variables to be more focused on those with a high degree of importance [61]. Moreover, random forest can handle high-dimensional data without the need for dimensionality reduction. It can still maintain accuracy when a large number of features are missing from the data. However, random forest still has many unsolved problems. For example, over-fitting may occur for some noisy classification or regression problems. In addition, too many variables will affect the accuracy of the results. To overcome the above shortcomings, scholars have refined the random forest method. Examples include Banzhaf random forests [62], weighted random survival forests [63], and quantile regression forests [64].

**Figure 2:**
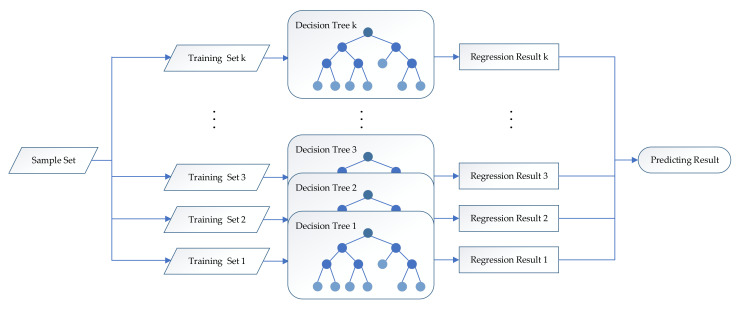
Random forest regression algorithm flow.

The main idea of random forest regression modeling is as follows. First, using a bagging method, *k* training sets are randomly selected from the sample set. Second, a decision tree model is constructed for each training set separately, and the training sets are assumed to be independently and identically distributed. Finally, a random forest regression model is formed by combining multiple decision trees. The prediction result of the model is the average of the regression results of the above *k* decision trees (as shown in Figure 2).

In random forest regression, the regression tree predicts the results as numerical values and assumes that the input and output vectors Y and X, respectively, of the training set are independently and identically distributed. The mean-squared generalization error of any numerical prediction h(x) is:

(1) $$E_{X,Y}{\left(Y\;-\;h\left(X\right)\right)}^{2}$$

For a random forest, the constituent base decision tree $\left\{h(x,\Theta_{k})\right\}$ is comprised of *k* decision trees. Random forest regression is obtained by taking the mean value from the decision trees with respect to *k*.

When the number of trees in a random forest grows infinitely, it is almost certain that,

(2) $$E_{X,Y}{\left(Y\;-\;av_{k}h\left(X,\Theta_{k}\right)\right)}^{2}\to E_{X,Y}{\left(Y\;-\;E_{\Theta }h\left(X,\Theta \right)\right)}^{2}$$

So, random forest regression formula is $Y\;=\;E_{\theta }h(X,\Theta )$. In practice, the approximate formula $Y\;=\;av_{k}h(X,\Theta_{k})$ is often used instead.

Express the right half of Equation (2) as the generalization error of the forest, which is $PE^{*}\left(forest\right)$. Define the generalization error of the decision tree as:

(3) $$PE^{*}\left(tree\right)\;=\;E_{\Theta }E_{X,Y}{\left(Y-h\left(X,\Theta \right)\right)}^{2}$$

Then,

(4) $$PE^{*}\left(forest\right)\;=\;E_{X,Y}{\left[E_{\Theta }\left(Y-h\left(X,\Theta \right)\right)\right]}^{2}\;=\;E_{\Theta }E_{\Theta \prime }E_{X,Y}\left(Y-h\left(X,\Theta \right)\right)\left(Y-h\left(X,\Theta \prime \right)\right)$$

Θ and Θ′ are independent of each other. The right-hand part of Equation (4) is the covariance, which can be written as: $E_{\Theta }E_{\Theta \prime }\left(\rho \left(\Theta ,\Theta \prime \right)sd\left(\Theta \right)sd\left(\Theta \prime \right)\right)$ in which $sd\left(\Theta \right)\;=\;E_{X,Y}\sqrt{{\left(Y\;-\;h\left(X,\Theta \right)\right)}^{2}}$. Define the weighted correlation coefficient as:

(5) $$\bar{\rho }\;=\;E_{\Theta }E_{\Theta \prime }(\rho (\Theta ,\Theta \prime )sd(\Theta )sd(\Theta \prime ))/{\left(E_{\Theta }sd(\Theta )\right)}^{2}$$

So,

(6) $$PE^{*}(forest)=\bar{\rho }{\left(E_{\Theta }sd(\Theta )\right)}^{2}\leq \bar{\rho }PE^{*}(tree)$$

The marginal effect of variable $X^{j}$ is a partial integral of the random forest as an empirical mean, which is the approximate expectation of all variables except $X^{j}$ [65]:

(7) $${\tilde{f}}_{j}\left(x\right)\;=\;\frac{1}{n}\sum_{i\;=\;1}^{n}{\hat{h}}_{RF}\left(x_{i}^{1},\ldots ,x_{i}^{j\;-\;1},x,x_{i}^{j\;+\;1},\ldots ,x_{i}^{p}\right)$$

As a result, we used a regression tree based random forest model approach for modeling, and the model setup is shown below:

(8) CEE~RED + FIN + IS + ER + OPEN + GOV + EI + FDI

The right end of the “~” in Equation (8) is the predictor variable of the model (the explanatory variable), and the left end is the response variable (the explained variable).

### 3.2. Description of Variables and Data

#### 3.2.1. Explained Variable

**Figure 3:**
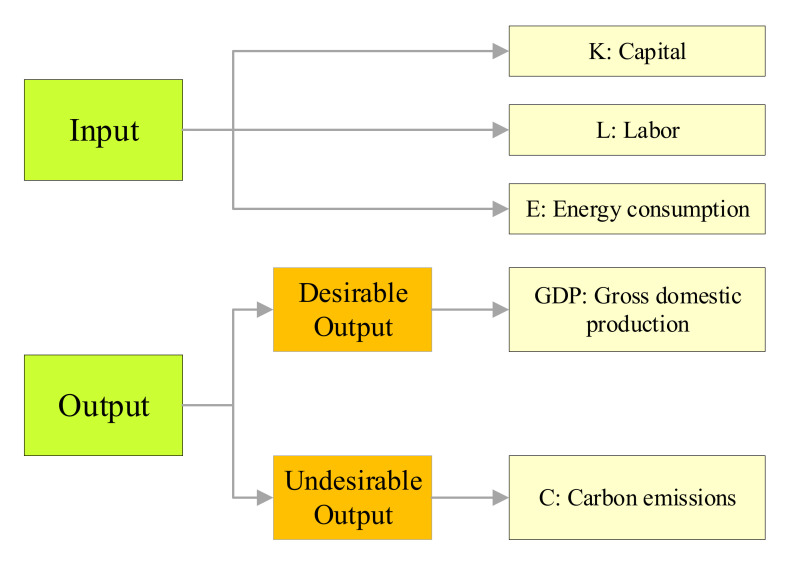
Indicators of carbon emission efficiency.

This study used carbon emission efficiency (CEE) as the explanatory variable. Measuring CEE requires the identification of inputs, desired outputs, and non-desired outputs. Inputs: capital stock (*K*), labor (*L*), and total energy consumption (*E*). Desired output: real gross domestic product (GDP). Non-desired output: carbon emissions (CO_2_). Among them, the capital stock *K* was measured by the perpetual inventory method with reference to the method of Zhang et al. [66,67], and its basic formula is: $K_{t}\;=\;I_{t}\;+\;\left(1-\delta_{t}\right)K_{t-1}$, where $K_{t}$ is the capital stock in year *t*, $I_{t}$ is the investment in year t, and $\delta_{t}$ is the depreciation rate in year *t*, formulated as 9.6%. The rest of the indicators can be found in the Statistical Yearbook (see Figure 3).

The calculation of CO_2_ was based on the methodology recommended in the 2006 IPCC Guidelines for National Greenhouse Gas Inventories (hereinafter referred to as “the Guidelines”) of the United Nations Intergovernmental Panel on Climate Change (IPCC) and is based on the following formula:

(9) $$CO_{2}\;=\;\sum_{all\;fuels}\left[\begin{array}{l} ((Apparent\;Consumption_{fuel}•Conv\;Factor_{fuel}•CC_{fuel})•10^{-3}\\ -Excluded\;Carbon_{fuel})•COF_{fuel}•(44/12) \end{array}\right]$$

It is worth noting that the Guidelines specifically emphasize that their conversion factors may not be representative of the country, which can cause bias in the calculation. After referring to the domestic carbon emission measurement methods, the Guidelines, combined with the China Energy Statistics Yearbook, were chosen to calculate China’s provincial carbon emissions with the following formula [68].

(10) $$CO_{2}\;=\;\sum_{i\;=\;1}^{8}E_{i}\times A_{i}$$

(11) $$A_{i}\;=\;a_{i}\times H_{i}\times \left(44/12\right)$$

where *CO*_2_ is the carbon dioxide emissions (unit: million tons), i is the energy type, which was taken from the China Energy Statistical Yearbook for the eight fossil energy sources other than electricity (coal, coke, crude oil, fuel oil, gasoline, kerosene, diesel, and natural gas), $E_{i}$ is the local consumption of energy i in 10^4^ t or 10^8^ m, $A_{i}$ is the CO_2_ emission rate of directly usable energy i in 10^4^ t/10^4^ t (i.e., 10,000 tons of carbon/ton of energy) or 10^4^ t/10^8^ m^3^ (i.e., 10,000 tons of carbon/billion cubic meters), which needed to be recalculated based on the net heating value calculation of each fuel type in the China Energy Statistics Yearbook, $a_{i}$ is the default emission factor for energy i provided in the Guidelines for i in kg/gj, $H_{i}$ is the net heating value of fuel i in a country or a region in kj/kg or kj/m^3^, and 44/12 is the molecular weight ratio of CO_2_ to C.

#### 3.2.2. Explanatory Variable

Renewable energy development (RED) was used as the core explanatory variable. According to the China Electricity Yearbook, we defined the following types of renewable energy sources: hydro, nuclear, wind, and solar. We used the ratio of the installed renewable energy capacity to the total installed capacity as a proxy variable for renewable energy development.

#### 3.2.3. Control Variables

To avoid omitting variables that cause endogeneity problems, we drew on previous studies to select the following control variables:

(1) Financial development (FIN). The impact of financial development on carbon emissions is consistent with the law of ‘short-term pain, long-term gain’ [69]. Financial development can improve the efficiency of the allocation of financial assets and can increase the inflow of intermediate goods to final goods when increasing the scale of production. This affects the carbon emission efficiency in the process [70].
(2) Industrial structure (IS). China needs to change its industrial structure by focusing on industrial groups with linkage characteristics if it wants to reduce CO_2_ emissions [71]. According to the statistics, industrial carbon emissions account for more than 70% of the total carbon emissions in China, so optimizing the industrial structure and actively developing the tertiary industries will have a direct impact on the carbon emission efficiency [72,73].
(3) Environmental regulation (ER). Environmental policies can produce significant adjustments in terms of the energy structure, industrial structure, and technological innovation [73,74]. Therefore, for energy-intensive industries, environmental regulation can promote CO_2_ emissions reductions and, thereby, have an impact on the carbon emission efficiency [75].
(4) Openness (OPEN). Foreign trade has led to a rapid increase in unjustified carbon emissions [76]. The development of foreign trade causes an economic transfer and also a pollution transfer, which in turn inevitably leads to a carbon emission spillover, both of which have an impact on the carbon emission efficiency at the same time [77].
(5) Government size (GOV). The expansion of the government’s size due to inter-governmental competition will, on the one hand, lead to the deterioration of the quality of the environment in a region, and, on the other hand, give the government the ability to deploy more financial resources to promote regional economic development [78].
(6) Energy intensity (EI). Currently, China’s energy use is still dominated by fossil energy, which may be the main reason for China’s carbon emissions. Energy intensity is an important indicator by which to measure energy efficiency [79], so a decrease in energy intensity means an increase in energy efficiency, which is the main reason for a reduction in carbon emissions [80].
(7) Foreign direct investment (FDI). Foreign direct investment can directly influence the country’s production, which affects both economic development and carbon emissions to some extent. At the same time, foreign investment will improve the country’s level of advanced technology and management style, which indicates that there is a spillover effect of FDI on the carbon emission efficiency [72].

The definition of these variables is shown in Table 2.

#### 3.2.4. Data

Panel data on 30 provinces, autonomous regions, and municipalities directly under the Central Government of China from 2006 to 2018 were used as the research sample. The data sources were the China Statistical Yearbook, the China Energy Statistical Yearbook, the China Electricity Yearbook, the China Population and Employment Statistical Yearbook, Provincial and Municipal Statistical Yearbooks, and the Wind database. All the nominal variables were price-deflated at constant 2000 prices. Table 3 shows the descriptive statistics for each variable.

## 4. Empirical Results and Discussion

### 4.1. Empirical Results

#### 4.1.1. Carbon Emission Efficiency

Based on the DEA game cross-efficiency model, we calculated the carbon emission efficiency of 30 provinces, autonomous regions, and municipalities from 2006 to 2018. The specific data for each year is shown in Appendix A Table A1. The average carbon emission efficiency of Beijing, Guangdong, Shanghai, Zhejiang, and Fujian ranked in the top five, while that of Ningxia, Qinghai, Shanxi, Xinjiang, and Inner Mongolia ranked in the bottom five, which shows that the carbon emission efficiency of the economically developed regions is relatively high.

**Figure 4:**
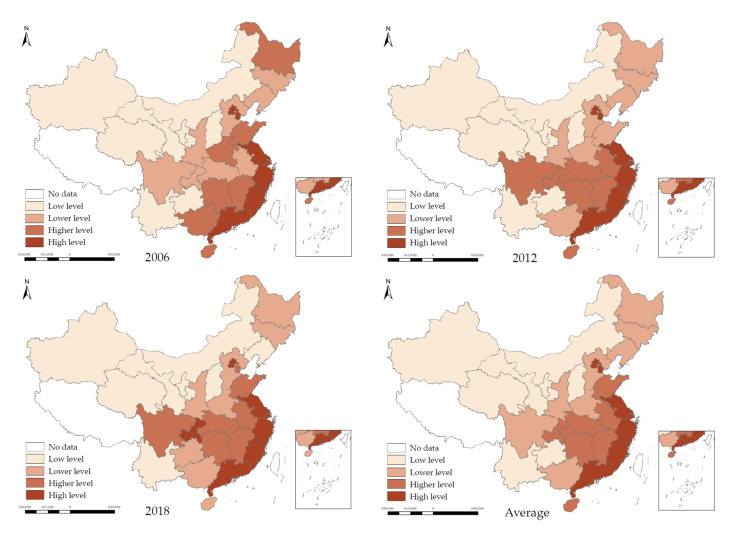
Classification of carbon emission efficiency in China in 2006, 2012, 2018 and annual average.

Using the quantile method, the carbon emission efficiency was then divided into four categories, namely high level, higher level, lower level, and low level, with darker colors indicating higher carbon emission efficiencies (Figure 4).

In Figure 4, there is the characteristic of a continuous distribution of regional carbon emission efficiency, with the east higher than the west and the south higher than the north. In particular, six provinces and cities, namely Beijing, Jiangsu, Shanghai, Zhejiang, Fujian, and Guangdong, were consistently classified at high levels. In contrast, Ningxia, Qinghai, Shanxi, Xinjiang, Inner Mongolia, Gansu, and Yunnan were consistently classified at low levels. From a temporal perspective, there was basically no significant change in the carbon emission efficiency of each province, and there was no jump across multiple categories.

#### 4.1.2. Empirical Analysis of the Random Forest Model

**Figure 5:**
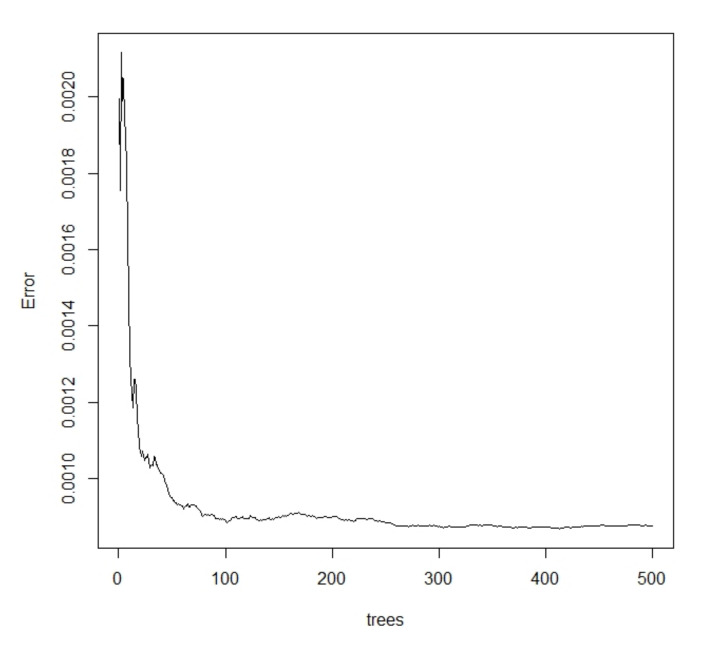
Error graph.

According to Equation (8), the ideal state is reached by using the bootstrap method with put-back. We used repeated sampling 300 times without pruning, the stopping rule was limited to include at least five sample points per node, and we used four randomly selected independent variables per tree. As shown in Figure 5, as the forest size increases, the prediction error rapidly decreases.

**Figure 6:**
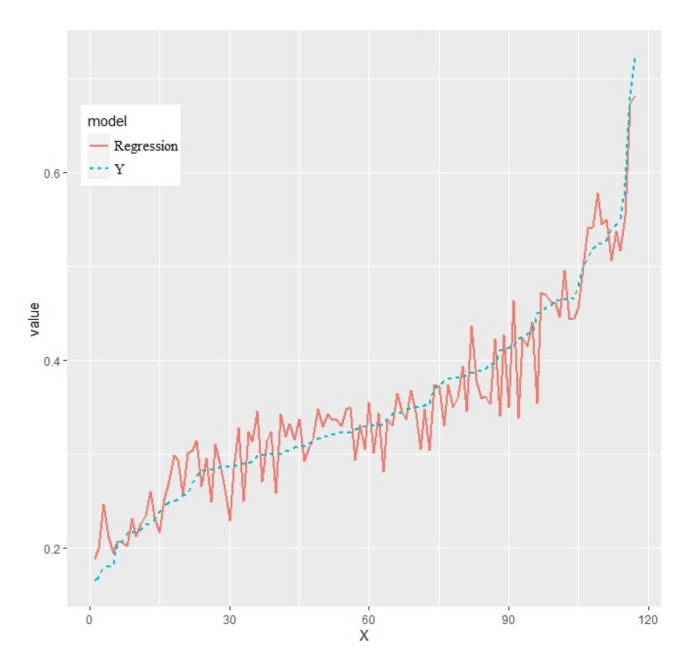
Test set results. Note: “X” denotes the number of regression results; “Y” denotes the actual values; “Regression” denotes the predicted values.

From Figure 5, it can be seen that the error from Equation (8) tends to be balanced when the forest size reaches about 300, and there is an increasing trend over 330. Therefore, the forest size chosen for this study was 300. The *R*^2^ is 91.66%, which indicates that the model has good explanatory strength. Using the test set to predict the random forest regression model, the root mean square error is 0.000391, which indicates that the prediction results are good and there is no overfitting. The predicted values of the random forest model on the test set were compared to the actual values (Figure 6). In Figure 6, the blue dashed line is the actual value of the carbon emission efficiency, and the red solid line is the predicted value of the random forest regression. The predicted value is consistent with the trend of the actual value and fluctuates around the actual value, which indicates that the prediction result is accurate.

**Figure 7:**
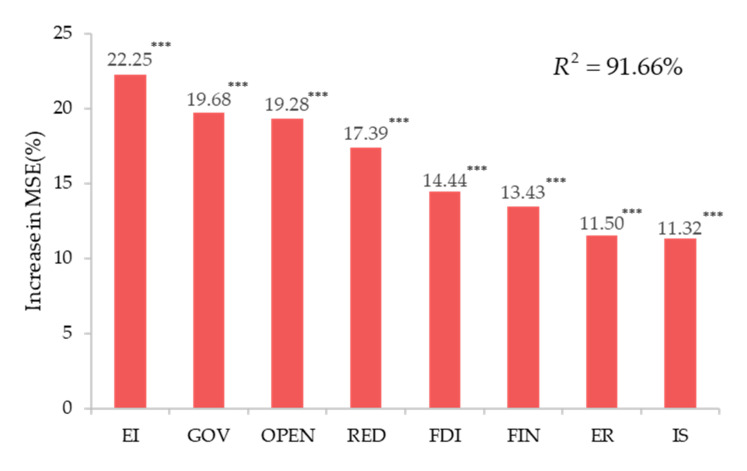
Importance and significance of each variable. Note: *** Significant at 1%.

Next, the importance of each predictor variable was assessed, and its significance was obtained. Since the data did not always satisfy the statistical tests, referring to the method of Jiao et al. [81], we used the principle of the permutation test to test the significance level. This was measured using the “increase in MSE % (i.e., percentage of increase in the mean square error)” value in the random forest model, where a higher MSE % value implies a more significant variable. Figure 7 shows the importance and significance of each variable.

As can be seen from Figure 7, EI, GOV, OPEN, and RED have a great impact on the carbon emission efficiency, and FDI, FIN, ER, and IS have a particular impact on the carbon emission efficiency. All variables are significant at the 0.01 level.

**Figure 8:**
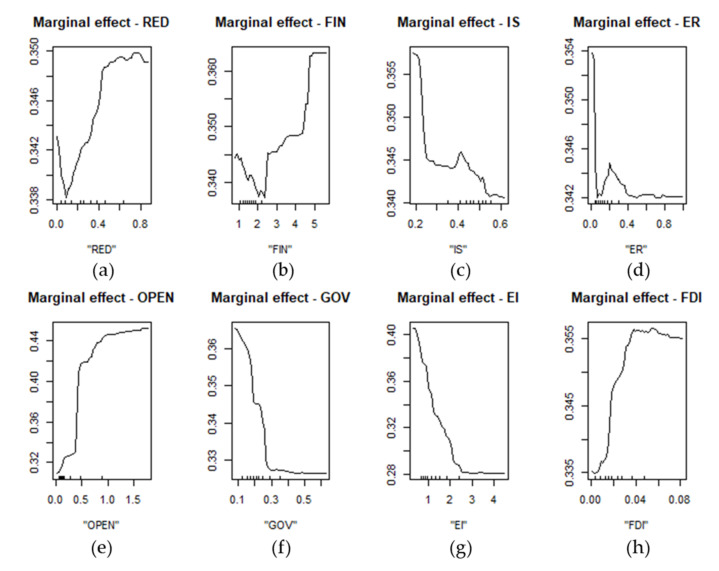
The marginal effect curve of each variable. Note: Figure (**a**–**h**) are marginal effect curves of RED, FIN, IS, ER, OPEN, GOV, EI and FDI on the carbon emission efficiency respectively. The specific definition of these variables is shown in Table 2.

The marginal effect curves of each variable on the carbon emission efficiency are shown in Figure 8. The increasing curve indicates that an increase in the variable helps to increase the carbon emission efficiency, and, in the converse situation, it hinders the carbon emission efficiency. The level of the marginal effect curve indicates that an increase or decrease in the variable has no effect on the carbon emission efficiency. The marginal effect curve illustrates that during the early stage of renewable energy development, when its proportion of the total energy is small, the development of renewable energy will make the carbon emission efficiency decrease. This effect will persist for a period of time until the renewable energy development reaches a certain level, at which point the renewable energy development will be conducive to the promotion of the carbon emission efficiency. This means that the upfront material and technical costs are the limitations that prevent the development of renewable energy from reaching its optimum level. When we combine this with each province’s current economic development characteristics, it can be concluded that the reasonable range of installed renewable energy capacity to total installed capacity is 35–55%. This conclusion is consistent with China’s goal of “a carbon peak by 2030 and carbon neutrality by 2060”. For the control variables, FIN, OPEN, and FDI are generally conducive to the improvement of the carbon emission efficiency, and their reasonable ranges are 4.5–5.05, 0.45–0.55, and 0.015–0.035, respectively. IS, ER, GOV, and EI are negatively related to the carbon emission efficiency, and their reasonable ranges are 0–0.15, 0.1–0.2, 0–0.1, and 0–0.35, respectively.

#### 4.1.3. Regional Heterogeneity Analysis

**Figure 9:**
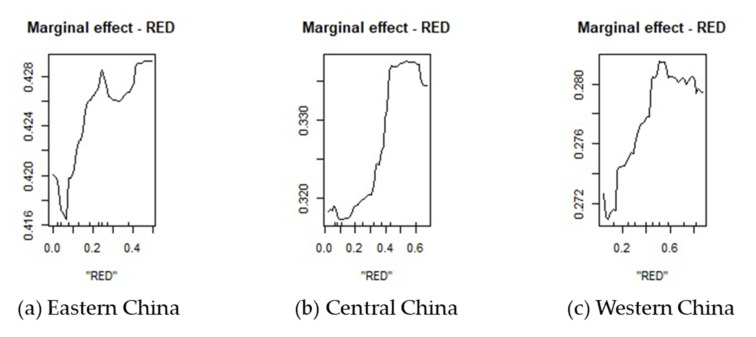
The marginal effect curve of RED in eastern, central, and western China. Note: Figure (**a**–**c**) are marginal effect curves of RED on the carbon emission efficiency in eastern, central, and western China respectively.

**Figure A1:**
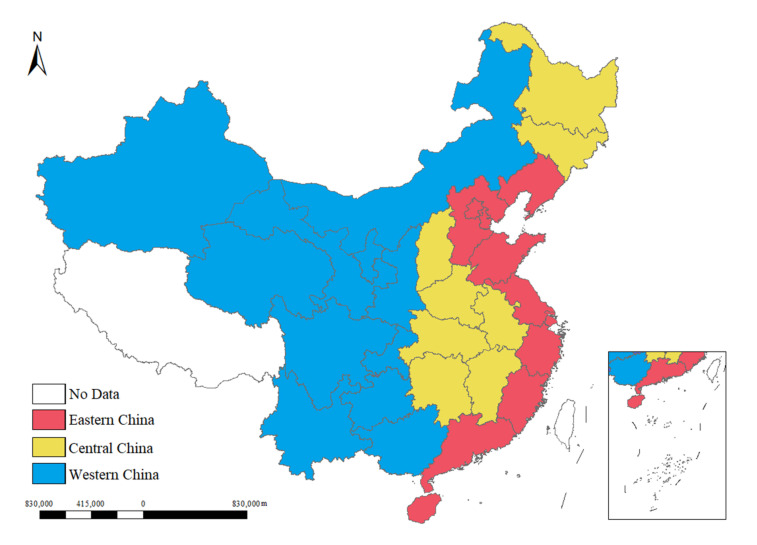
Three major regions: eastern China, central China, and western China.

It is necessary to further explore the impact of regional renewable energy development on carbon emission efficiency due to China’s vast territory and resources. Different regions have different resource endowments and economic development levels. We divided China into eastern, central, and western regions (Appendix A Figure A1) for a regional heterogeneity analysis. Figure 9 shows the marginal curves for each prediction variable of the random forest regression in the eastern, central, and western regions, respectively.

As can be seen from Figure 9, the impact trend of renewable energy development on the eastern, central, and western regions is to first inhibit, second promote, then reach a reasonable range, and finally decline. However, the reasonable range of the three regions is different; the highest range is in the western region (0.42–0.79), the second-highest range is in the central region (0.40–0.60), and the lowest range is in the eastern region (0.20–0.50). The reasonable ranges of carbon emission efficiency corresponding to the reasonable ranges of renewable energy development in the three regions are: greater than 0.426 in the east, greater than 0.334 in the central region, and greater than 0.280 in the west.

### 4.2. Discussion

The carbon emission efficiency was found to be higher in the east than in the west, and higher in the south than in the north, which is consistent with the findings of Cheng et al. [38] and Wang et al. [78]. The northeastern region, which has the largest industrial base in China, has a long history of high energy consumption and high carbon emissions. Still, its economic development is slow, and its carbon emission efficiency continues to decline. The western region has a lower technology level and a low output-input ratio, and, although its carbon emissions are lower, its carbon emission efficiency is at a lower level. The eastern region has a high level of technological productivity, a high level of economic development, and environmental policies that are focused on the eastern region, resulting in the carbon emission efficiency in these provinces being at nationwide high levels.

Renewable energy has a scale effect. With the increase in investment in renewable energy, the impact on the carbon emission efficiency is restrained first and then promoted. In 2018, the development of renewable energy in the Sichuan, Yunnan, and Qinghai provinces reached a reasonable range. However, only Sichuan province reached a reasonable range of carbon emission efficiency in 2018, which means that developing renewable energy is not the only way to improve carbon emission efficiency. Cooperation and efforts in many aspects are required to improve the carbon emission efficiency, which is consistent with the findings of Liu et al. [16].

It is worth noting that the order of the reasonable range of renewable energy development in the eastern, central, and western regions is opposite to the corresponding optimal range of carbon emission efficiency. The eastern region, with the lowest reasonable range of renewable energy development, has the highest optimal carbon emission efficiency. In contrast, the western region, with the highest reasonable range, has the lowest optimal carbon emission efficiency.

We speculate that there may be two reasons for this phenomenon. First, there are differences in the natural environments of the three regions in terms of geographical location, climate, and resource endowment. Second, there are differences in the human activities in the three regions, which are reflected in the economy, society, and culture. For example, the western region has strong sunlight and a large geographical drop, which makes it easier to generate solar power, hydropower, and onshore wind power. The eastern region has a broad coastline, so offshore wind power generation and tidal power generation are more suitable. In addition, due to the unbalanced allocation of educational resources, the education level in the eastern region is significantly higher than that in the central and western regions, resulting in significant differences in the quality of human resources among the regions [82].

Based on the above results, we have drawn the following conclusions.

Firstly, energy transition has a positive effect in the long run, which suggests that ultimately the returns from energy transition outweigh its costs. This is in line with the assumptions made about energy transition in this study. The use of renewable energy has been shown to reduce environmental pollution and to contribute to economic development, technological progress, and industrial upgrade [83]. To promote energy transition, China has established demonstration cities for energy transition and renewable energy development and has proven that this policy is effective [84]. However, to ensure the sustainability of energy transition policies, it is important to be prepared for possible periods of pain. This is because, in the short term, energy transition can have a negative impact due to the imperfections in the existing system, which can undermine the initial policy ambitions and cause hardship during the transition period [85].

Secondly, there is a threshold for the benefits brought about by the development of renewable energy, and promotion will only occur after a certain level of investment in renewable energy is reached. During the early stage of renewable energy development, a large amount of infrastructure construction has to be carried out, which requires human and capital investment. For these investments, the return is not immediately realized, and must increase with the investment to a certain level before a positive return is achieved [86]. This also illustrates that a natural threshold is formed during the early stages of energy transition due to the high cost of renewable energy and increased carbon emissions [87]. This suggests that the energy transition is not solely related to the energy endowment and policies [84].

Finally, the development of renewable energy in the eastern region produced effects more quickly. Carbon emission efficiency is not only related to the carbon emissions of a region; it is also highly correlated to the economic development of that region. The eastern region’s higher carbon emissions indicate a greater potential for carbon emissions reductions and a more rapid response to the energy transition. Moreover, its higher level of economic development may enable it to provide more financial support [88] and more professionals [89], providing a constant supply of fresh blood for renewable energy development. For the western region, both the carbon emissions and the economic development levels are lower. This makes energy transition policies take effect more slowly. However, because of its larger renewable energy endowment, it has a natural advantage in the development of renewable energy. More consideration needs to be given to the question of how to sustainably develop renewable energy while ensuring long-term economic development.

## 5. Conclusions

Using 2006–2018 as the base period, this study explored the impact of renewable energy development on carbon emission efficiency and the impact paths in 30 Chinese provinces, municipalities, and autonomous regions. The carbon emission efficiency was measured with a game cross-efficiency model considering non-desired outputs. A random forest model was used to regress the renewable energy development and carbon emission efficiency. Finally, China was divided into three regions (eastern, central, and western) to discuss the impact of regional differences on the study results. The main findings of this study are as follows.

(1) The impact of renewable energy development on carbon emission efficiency is very important and significant. It mainly shows a trend of inhibition during the early stage and promotion later, when an obviously reasonable range is reached.
(2) Energy intensity, foreign trade, and government size are the three most prominent factors influencing carbon emission efficiency. They all have a greater influence than renewable energy development. Their influence directions are roughly negative, positive, and negative, respectively.
(3) Through the regional heterogeneity analysis, it was found that there is no difference in the trend of the influence of renewable energy development on the carbon emission efficiency. Still, there was a great difference in the values, especially in the reasonable range. The eastern region has the lowest reasonable range of renewable energy development but the highest reasonable range of carbon emission efficiency; the western region has the highest reasonable range of renewable energy development but the lowest reasonable range of carbon emission efficiency.

### 5.1. Policy Implications

According to the above findings, we propose several policy implications.

(1) Encourage technological innovation and promote the sustainable development of energy transition.

Long-term results show that the energy transition will have a positive impact on the economy and the environment. However, in the short term, due to imperfections in objective factors, the energy transition will still create temporary dilemmas. To actively address the negative short-term impacts of the energy transition, it is necessary to promote the energy transition while ensuring economic growth. Technological innovation is the key. On the one hand, the government needs to strengthen the guidance on technological innovation and actively promote the close cooperation of organizations throughout the whole industry chain and vertical cooperation to overcome technical difficulties. On the other hand, it also needs to give greater financial support to renewable energy enterprises with a large proportion of technical investment and encourage these enterprises to conduct technical exchanges and share technology to improve the technology level of the whole industry as soon as possible.

(2) Persistent development of renewable energy.

The marginal impact modeling results show that the development of renewable energy will inhibit the improvement of the carbon emission efficiency during the early stage, but this does not prove that developing renewable energy is not worthwhile. What might cause this is the necessity of providing certain inputs during the early stage of the development of renewable energy, such as the construction of renewable energy power generation equipment, which has no positive return in the short term, so it reduces the carbon emission efficiency. During the middle stage of renewable energy development, the carbon emission efficiency continues to rise, which indicates that the renewable energy development begins to play a positive role. During the later stages of renewable energy development, after a reasonable amount of time, the carbon emission efficiency will reach a relatively optimal value and a reasonable range, which will bring the renewable energy development to a satisfactory level. The vast majority of energy consumed is renewable energy.

(3) Formulate targeted renewable energy development strategies according to local conditions.

For the western regions with a high reasonable range of renewable energy and a low reasonable range of carbon emission efficiency, it is necessary to promote local economic development while encouraging the development of renewable energy. In contrast, for the eastern regions, the government should pay attention to the protection of the environment in the process of economic development. In addition, due to the differences in the distribution of resource endowments, it is necessary to strengthen the cooperation between the east and the west. For example, renewable energy developed in the west can be transmitted to the east through the national grid, which may supplement the shortage of renewable energy production in the east and generate income for the west.

### 5.2. Outlook

Our study has some limitations arising from the complexity of the relationship between renewable energy development and carbon emission efficiency. The first is that the measure of provincial renewable energy development is relatively simple, and this could be improved in the future by enhancing the quality of the data. The second is that the marginal impacts measured by the random forest regression model are based on the existing data, so we can only derive the magnitude of impacts, trends in impacts, and reasonable ranges under the existing development conditions; this study did not have a predictive function.

In the future, this work will be improved in several respects. First, we will explore the use of better data and models, and we will study the renewable energy development and carbon emission efficiency of different countries. Comparisons will be made between developed and developing countries, and we will explore the renewable energy development methods suitable for countries with varying levels of development. Second, a sustainable energy transition requires multiple types of support, and financial support is essential. Green finance can facilitate the process of energy transition [90], and it is necessary to explore the transmission model. Finally, energy transition policies will inevitably be implemented in specific industries, such as the automotive industry, which is already implementing vehicles that use new types of energy [91]. Therefore, it is also important to consider the impact of the energy transition on different industries and how it will affect them.

## Figures and Tables

**Table 1:** Studies measuring carbon emission efficiency.

Literature	Method	Input	Desirable Output	Undesirable Output
Zeng et al. [39]	EBM-DEA	Capital stock Labor Energy consumption	Real GDP	CO_2_ emissions
Xie et al. [40]	Super-SBM	Capital stock Labor Energy consumption	Real GDP	CO_2_ emissions
Du et al. [31]	Super-SBM	Capital stock Labor Energy consumption Machines	Industrial economic output	CO_2_ emissions
Li et al. [41]	Three-stage DEA	Capital stock Labor Energy consumption	Real GDP	CO_2_ emissions
Zhang et al. [42]	Three-stage DEA	Capital stock Labor Energy consumption	Real GDP	CO_2_ emissions

**Table 2:** Definition of research variables.

Variables	Definition
CEE	Carbon emission efficiency
RED	Percent of installed renewable energy capacity in the total installed capacity
FIN	Percent of balance of deposits and loans in GDP
IS	Percent of secondary industry in GDP
ER	Percent of industrial pollution control investment in GDP
OPEN	Proportion of total export–import volume in GDP
GOV	Percent of government expenditure in GDP
EI	Percent of total energy consumption in GDP
FDI	Percent of foreign direct investment in GDP

**Table 3:** Descriptive statistics of variables.

Variables	Number	Mean	Std. Dev	Min	Max
CEE	390	0.3448	0.1072	0.1545	0.7640
RED	390	0.3026	0.2231	0.0008	0.8679
FIN	390	1.6898	0.7390	0.8186	5.5866
IS	390	0.4612	0.0832	0.1863	0.6148
ER	390	0.1500	0.1337	0.0067	0.9918
OPEN	390	0.3031	0.3656	0.0164	1.7705
GOV	390	0.2260	0.0971	0.0830	0.6269
EI	390	1.3315	0.7134	0.3256	4.4715
FDI	390	0.0230	0.0179	0.0004	0.0819

## Data Availability

Data available in a publicly accessible repository. The data presented in this study are openly available in China Statistical Yearbook, China Energy Statistical Yearbook, China Electricity Yearbook, China Population and Employment Statistical Yearbook, provincial and municipal Statistical Yearbooks, and the Wind database.

## Appendix A

**Table A1:** The carbon emission efficiency of Chinese provinces from 2006 to 2018.

**Province**	**2006**	**2007**	**2008**	**2009**	**2010**	**2011**	**2012**
Beijing	0.5382	0.5188	0.5095	0.5279	0.5242	0.5901	0.6280
Tianjin	0.4622	0.4489	0.4218	0.4179	0.3994	0.4041	0.4277
Hebei	0.3482	0.3115	0.2727	0.2517	0.2600	0.2766	0.3082
Shanxi	0.2654	0.2262	0.1892	0.1801	0.1964	0.2123	0.2315
Inner Mongolia	0.3086	0.2672	0.2330	0.2450	0.2372	0.2385	0.2565
Liaoning	0.3485	0.3107	0.2861	0.2758	0.2867	0.3020	0.3316
Jilin	0.3543	0.3315	0.3129	0.2937	0.2891	0.3029	0.3261
Heilongjiang	0.4071	0.3651	0.3234	0.2924	0.2939	0.3076	0.3316
Shanghai	0.5220	0.4991	0.4648	0.4652	0.4455	0.4609	0.5027
Jiangsu	0.4643	0.4338	0.4092	0.3914	0.3870	0.4079	0.4517
Zhejiang	0.4973	0.4580	0.4355	0.4052	0.4083	0.4366	0.4791
Anhui	0.3781	0.3516	0.3219	0.3123	0.3227	0.3423	0.3814
Fujian	0.5017	0.4639	0.4440	0.4209	0.4118	0.4298	0.4657
Jiangxi	0.4020	0.3641	0.3400	0.3481	0.3474	0.3712	0.4075
Shandong	0.3976	0.3729	0.3471	0.3232	0.3199	0.3236	0.3534
Henan	0.3852	0.3500	0.3248	0.2925	0.2905	0.2885	0.3214
Hubei	0.3454	0.3453	0.3229	0.3084	0.3167	0.3305	0.3687
Hunan	0.3817	0.3510	0.3175	0.3076	0.3170	0.3385	0.3786
Guangdong	0.6088	0.5786	0.5396	0.5116	0.5051	0.5287	0.5616
Guangxi	0.4194	0.3989	0.3806	0.3291	0.3180	0.3298	0.3459
Hainan	0.4576	0.3841	0.3508	0.3377	0.3430	0.3495	0.3761
Chongqing	0.3370	0.3022	0.2745	0.3007	0.3062	0.3445	0.3872
Sichuan	0.3516	0.3278	0.2879	0.2734	0.2908	0.3237	0.3675
Guizhou	0.2245	0.2055	0.1933	0.1953	0.2024	0.2282	0.2704
Yunnan	0.2999	0.2834	0.2669	0.2438	0.2443	0.2626	0.2938
Shaanxi	0.3257	0.3088	0.2863	0.2844	0.2844	0.3042	0.3295
Gansu	0.2869	0.2663	0.2389	0.2227	0.2327	0.2473	0.2799
Qinghai	0.2349	0.2250	0.2003	0.1959	0.2077	0.2100	0.2221
Ningxia	0.1940	0.1778	0.1582	0.1632	0.1702	0.1838	0.2022
Xinjiang	0.2895	0.2629	0.2308	0.2017	0.2164	0.2201	0.2362
**Province**	**2013**	**2014**	**2015**	**2016**	**2017**	**2018**	**Average**
Beijing	0.6667	0.6749	0.6815	0.6860	0.7232	0.7640	0.6179
Tianjin	0.4413	0.4302	0.4019	0.3970	0.4114	0.4160	0.4215
Hebei	0.3103	0.2992	0.2699	0.2585	0.2701	0.2912	0.2868
Shanxi	0.2203	0.2054	0.1836	0.1679	0.1973	0.2304	0.2081
Inner Mongolia	0.2612	0.2495	0.2185	0.2008	0.1793	0.1989	0.2380
Liaoning	0.3512	0.3441	0.3150	0.2353	0.2501	0.2896	0.3021
Jilin	0.3468	0.3385	0.3150	0.3042	0.2999	0.3133	0.3176
Heilongjiang	0.3478	0.3213	0.2829	0.2564	0.2739	0.2933	0.3151
Shanghai	0.5117	0.5369	0.5246	0.5280	0.5454	0.5837	0.5070
Jiangsu	0.4666	0.4653	0.4566	0.4516	0.4764	0.5059	0.4437
Zhejiang	0.4871	0.4816	0.4631	0.4569	0.4741	0.5028	0.4604
Anhui	0.3804	0.3829	0.3568	0.3520	0.3640	0.3919	0.3568
Fujian	0.4805	0.4621	0.4432	0.4339	0.4503	0.4793	0.4529
Jiangxi	0.4186	0.4171	0.3878	0.3818	0.3893	0.4160	0.3839
Shandong	0.3747	0.3701	0.3425	0.3312	0.3439	0.3639	0.3511
Henan	0.3272	0.3261	0.3004	0.2932	0.3015	0.3211	0.3171
Hubei	0.3995	0.3994	0.3722	0.3615	0.3705	0.4000	0.3570
Hunan	0.3960	0.3910	0.3622	0.3528	0.3602	0.3900	0.3572
Guangdong	0.5682	0.5560	0.5258	0.5100	0.5307	0.5498	0.5442
Guangxi	0.3546	0.3498	0.3304	0.3184	0.3142	0.3458	0.3488
Hainan	0.3814	0.3685	0.3279	0.3189	0.3300	0.3431	0.3591
Chongqing	0.4218	0.4248	0.4007	0.4109	0.4226	0.4501	0.3679
Sichuan	0.3832	0.3774	0.3487	0.3534	0.3724	0.4132	0.3439
Guizhou	0.2866	0.2992	0.2872	0.2757	0.3061	0.3329	0.2544
Yunnan	0.3027	0.2987	0.2738	0.2558	0.2630	0.2719	0.2739
Shaanxi	0.3304	0.3210	0.2835	0.2698	0.2978	0.3257	0.3040
Gansu	0.2820	0.2826	0.2466	0.2357	0.2478	0.2833	0.2579
Qinghai	0.2153	0.2057	0.1829	0.1685	0.1619	0.1739	0.2003
Ningxia	0.2017	0.1917	0.1674	0.1549	0.1545	0.1744	0.1765
Xinjiang	0.2324	0.2166	0.1884	0.1654	0.1819	0.2065	0.2191
